# Phytyl Phenolipids: Structurally Modified Antioxidants with Superior Lipid Membrane Interaction

**DOI:** 10.3390/molecules30102193

**Published:** 2025-05-17

**Authors:** Vânia Costa, Marlene Costa, Rute Rebelo, Francisca Arques, Mariana Ferreira, Paula Gameiro, Tomás Barros, Dulce Geraldo, Luís S. Monteiro, Fátima Paiva-Martins

**Affiliations:** 1 Associated Laboratory for Green Chemistry (LAQV) of the Network of Chemistry and Technology (REQUIMTE), Department of Chemistry and Biochemistry, Faculty of Sciences, University of Porto, 4169-007 Porto, Portugal; vaniasilvacosta@gmail.com (V.C.); marlene.andreia.costa@gmail.com (M.C.); rutesararebelo@gmail.com (R.R.); up202007509@edu.fc.up.pt (F.A.); mariana.ferreira@fc.up.pt (M.F.); agsantos@fc.up.pt (P.G.); 2 Chemistry Centre, University of Minho (CQ-UM), Gualtar, 4710-057 Braga, Portugal; tomasaraujobarros@gmail.com (T.B.); gdulce@quimica.uminho.pt (D.G.); monteiro@quimica.uminho.pt (L.S.M.)

**Keywords:** phenolic acids, phenolipids, phytyl esters, liposomes, biomembrane, caffeic acid, homoprotocatechuic acid, protocatechuic acid, dihydrocaffeic acid, lipophenols

## Abstract

A set of procedures was developed for the simple synthesis of phytyl phenolipids, which resulted in high yields (70–95%) of phytyl esters of caffeic, protocatechuic, homoprotocatechuic, and dihydrocaffeic acids. Initial characterization revealed that these new compounds exhibited similar radical scavenging activity and liposolubility to α-tocopherol, a key antioxidant present in membranes. Cyclic voltammetry analysis indicated that the phytyl derivatives had lower anodic peak potentials compared to the original phenolic acids, with electron transfer following an adsorption-controlled mechanism. In phosphatidylcholine large unilamellar vesicles (LUVs), phytyl esters demonstrated remarkable efficiency in preventing liposome autoxidation when compared to α-tocopherol. Despite their strong radical scavenging capacity and membrane penetration ability, the antioxidant effectiveness of the phytyl esters in liposomes was influenced by the structure of their polyphenolic moiety. These new compounds are considered promising candidates for future pharmacological applications against oxidative stress in lipoproteins and cells, warranting further evaluation of their antioxidant and anti-inflammatory effects in cellular models and in vivo.

## 1. Introduction

Cardiovascular diseases (CVDs), hepatic steatosis, diabetes, and neurodegenerative diseases continue to affect large portions of the population, despite the many therapeutic treatments already developed [1,2,3,4,5]. The causes of these disorders are diverse and complex, but scientific evidence points to two common factors for these conditions: inflammation and oxidative stress [6,7,8,9]. Many well-known risk factors such as hypertriglyceridemia, hyperglycemia, high levels of glycated hemoglobin, and decreased high-density lipoprotein cholesterol (HDL-C) promote mitochondrial dysfunction, resulting in increased production of reactive oxygen species (ROS). On the other hand, associated with this increase in ROS, the inhibition of antioxidant defense systems, with emphasis on the reduction in the activity of antioxidant enzymes such as catalase (CAT), superoxide dismutase (SOD), and enzymes involved in glutathione metabolism (GSH) can also occur [6,7,8]. This oxidative stress not only activates several inflammatory factors, including C-reactive protein, inflammasome protein 3 (NLRP3), tumor necrosis factor alpha (TNFα), and nuclear factor kappa B (NF-kB) [6], but also increases low-density lipoproteins (LDL) and HDL oxidation together with endothelial dysfunction, favoring the formation of atheroma [9]. Given that metabolic diseases and aging are strongly related to oxidative stress [10], membrane-targeted antioxidants may help reduce oxidative damage to organelles, cells, and lipoproteins.

Plants are dynamic and aggressive living organisms focused on survival and reproduction, having the ability to recognize and respond to their own needs and external threats such as parasites and to changes in weather conditions. In order to fight oxidative stress caused by low water availability and sun exposure, plants are required to produce antioxidant defenses, namely to produce antioxidant molecules (AOs) [11]. Animals, on the other hand, are incapable of such biosynthesis and need to consume these molecules that will protect their cells. α-Tocopherol produced by plants has been used by animal cells as the most important AOs against membrane oxidative injury. Several classes of other phenolic antioxidant molecules such as flavonoids and phenolic acids have also been shown to play an important role in maintaining health and preventing chronic diseases by preserving the structural integrity of biomembranes and mitigating the effects of oxidative stress [11,12]. However, most of these phenolic molecules have been “designed” by plants for their own needs, being that their use by animals is limited by possible toxicity or by low bioavailability, mostly due to their hydrophilic nature [11,13].

The liposolubility of phenolic acids can be increased and, thus, improve their bioavailability through esterification with an alkyl chain. These new molecules, named phenolipids (or lipophenols), have the capacity to interact with and cross membranes, leading to a good pharmacological efficacy [3]. Caffeic, ferulic, and gallic acid phenolipids, depending on the length of the alkyl chain, have been shown to protect LDL from oxidative damage [14,15]. Some studies have also been conducted on caffeic acid phenolipids using red blood cells (RBCs), and a strong protective activity against 2,2′-azobis(2-amidinopropane) dihydrochloride (AAPH)-induced oxidative was observed at a concentration as low as 2.5 μM [16]. However, some studies using phenolipids have shown some toxic effects in cell models, in some cases in a very low concentration [17,18]. Either the phenolipid itself or its metabolization products can be responsible for this toxicity. Preliminary toxicological studies with some classes of phenolipids have shown that lipophilization using linear fatty alcohols are more likely to produce toxicity than using phytosterols, a more common kind of phenolic esters found in foods [18]. Therefore, in the search for more efficient AOs able to fight oxidative stress, particularly in the case of degenerative conditions, the design of molecules inspired on natural compounds already found in foods may be considered a safer approach.

**Figure 1:**
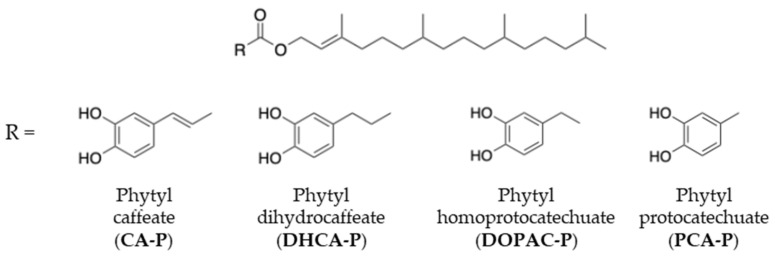
Synthetized phytol-derived phenolipids.

Phytol [(*E*)-3,7,11,15-tetramethylhexadec-2-en-1-ol], an acyclic monounsaturated diterpene alcohol, is an example of a potential natural compound to be used in pharmacological applications [19,20]. It constitutes a key structural component of chlorophyll and serves as the biosynthetic precursor of the phytane side chain in α-tocopherol—widely recognized as the most important antioxidant molecule in biomembranes and lipoproteins [11,19,20]. α-Tocopherol plays a crucial role in protecting cellular membranes from oxidative damage, and its universal presence in animal cells underscores its biological relevance. In both chlorophyll and α-tocopherol, nature employs the phytane chain to anchor these functionally critical molecules within lipid bilayers, precisely where antioxidant protection is most needed. Inspired by this natural strategy, the present work aimed to synthesize α-tocopherol analogs by conjugating the phytol chain to potent radical-scavenging polyphenolic acids—namely, caffeic acid (CA), dihydrocaffeic acid (DHCA), homoprotocatechuic acid (DOPAC), and protocatechuic acid (PCA). The resulting phenolipids are designed to act as lipophilic antioxidants, mimicking the membrane-targeting and protective functions of α-tocopherol, while potentially offering enhanced efficacy in the prevention or treatment of degenerative diseases associated with oxidative stress (Figure 1).

## 2. Results and Discussion

### 2.1. Synthesis of Phytyl Phenolipids

The esterification of polyphenolic acids often requires the previous protection of aromatic hydroxyl groups due to their particular chemical reactivity in base catalyst conditions, leading to time and material-intensive processes leading to low overall yields [13,21,22,23]. In this work, we used acidic conditions to avoid, on the one hand, the need of this protective step and, on the other hand, the easier oxidation of polyphenolic acids in basic media as already described in our previous work concerning caffeic acid esters and cholesterol-derived phenolipids [13]. However, under the same reaction conditions, the yields obtained were quite different depending on the phenolic acid structure and solvent. In toluene, no reaction was observed, but in THF, a moderate yield for DOPAC and a low yield for DHCA were obtained. In agreement with what had been observed for the synthesis of cholesteryl protocatechuate in our previous work [13], PCA was shown not to be reactive under acidic conditions in the presence of phytol (Table 1).

Since phytol is a primary alcohol, the esterification reaction under enzymatic catalysis was also attempted in several solvents. Good yields were obtained for phytyl dihydrocaffeate (DHCA-P) and phytyl homoprotocatechuate (DOPAC-P) (Table 1). The solvent was an important factor for success of the esterification, with toluene and dioxane favoring the reaction. The reaction also showed an important dependence on the structure of the polyphenol as the yields of DHCA-P and DOPAC-P were the highest in different solvents, namely toluene and dioxane, respectively. In contrast, the phytyl protocatechuate (PCA-P) was obtained in a modest yield and only when THF was used as solvent (Table 1).

Since the PCA-P was not obtained in good yields with the above synthetic approaches, PCA was made to react as a chloride with phytol. Therefore, the synthesis of PCA ester was achieved in good yields (70%) in a one-pot procedure by first reacting PCA with thionyl chloride in dichloromethane followed by the addition of phytol [13].

Phytyl caffeate (CA-P) was obtained in a good yield (95%) by a well-known procedure, with a two-step synthetic route in a one-pot synthesis, based on a Knoevenagel condensation [13].

In general, the reaction times were long; however, this drawback was surpassed by a reduction in synthetic steps and easier purification procedures.

### 2.2. Evaluation of Free Radical Scavenging Capacity of Phytyl Phenolipids

The free radical scavenging ability of antioxidants in solution was assessed using the 2,2-diphenyl-1-picrylhydrazyl (DPPH) radical assay [12,23]. Based on the EC50 values (Table 2), the incorporation of the phytyl group generally reduced the radical scavenging capacity of phytyl esters compared to the parent compounds, except for the PCA ester, which demonstrated improved activity. The addition of a bulky ester group like phytyl could create steric hindrance, hindering the interaction between the phenolic hydroxyl group and the DPPH radical. For the PCA phytyl ester, however, this steric hindrance may be offset by an increased reactivity of the phenolic hydroxyl group, resulting from the esterification of the carboxyl group directly attached to the aromatic ring, as previously noted for cholesteryl protocatechuate [12]. Despite these variations, under the tested conditions, phytyl phenolipids exhibited superior radical scavenging activity compared to α-tocopherol.

Since AOs react with free radicals by donating electrons or hydrogens, oxidation potential is a fundamental indicator of their possible effectiveness as AOs. Thus, cyclic voltammetry was used to determine the oxidation peak potential of each AO, providing valuable information about their susceptibility to oxidation (Table 3).

**Figure 2:**
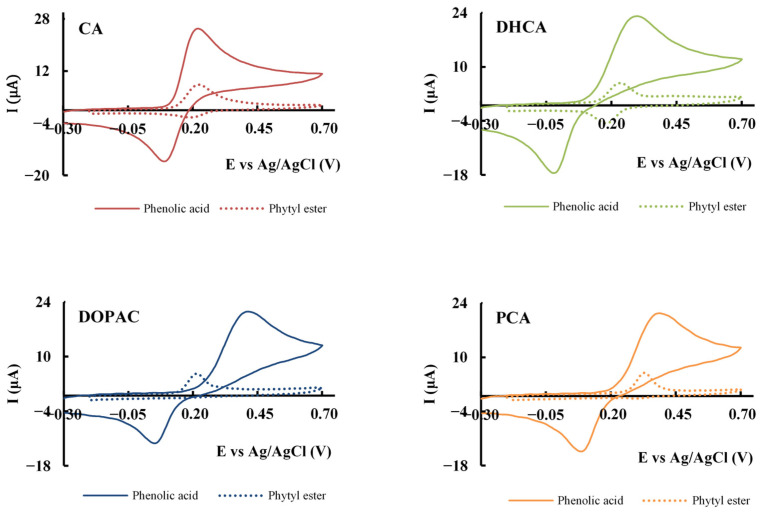
Comparison of the voltammetric response of phenolic acids (solid line) and their corresponding phytyl esters (dashed line). Cyclic voltammograms obtained in a phosphate buffer (pH 7.4) using a glassy carbon electrode (GCE) at 100 mV/s for 1 mM of compound.

The anodic peak potential of the AOs was used to characterize their potential antioxidant capacity and to determine the nature of the process controlling the oxidation reaction. A comparative analysis of the voltammograms for the phenolic acids and their corresponding phytyl esters offers insights into their redox behavior and stability (Figure 2).

Among the phenolic acids studied (Figure 2, Table 3), CA exhibits the lowest anodic peak potential (223 ± 4 mV), highlighting its superior propensity to oxidize and, thus, to have a potentially greater antioxidant activity. Although all AOs displayed low anodic peak potential values, α-Toc displayed the lowest (219 ± 2 mV).

In the case of phenolic acids, their electrochemical behavior is characterized by relatively low oxidation potentials, indicating high reactivity (Table 3). The presence of a cathodic peak in the reverse scan shows that the behavior of these compounds is reversible.

When these phenolic acids were esterified with phytol, notable changes were observed in their electrochemical behavior. All esters exhibited a lower oxidation peak potential compared to their respective parental compound, except for CA, which showed no significant variation when considering the standard deviation (235 ± 11 mV for CA-P and 223 ± 4 mV for CA). A lower anodic potential is beneficial and corresponds to an increased ability to donate electrons to free radicals. Esterification also leads to lower peak currents for all compounds, which can be attributed to a decrease in the diffusion coefficient of the species due to the higher molecular weight of these molecules. Except for DOPAC-P, all esters displayed cathodic peaks in the reverse scan, which indicates for these compounds that the processes are reversible (Figure 2). A lack of cathodic peak was also found for α-Toc in the same conditions.

**Figure 3:**
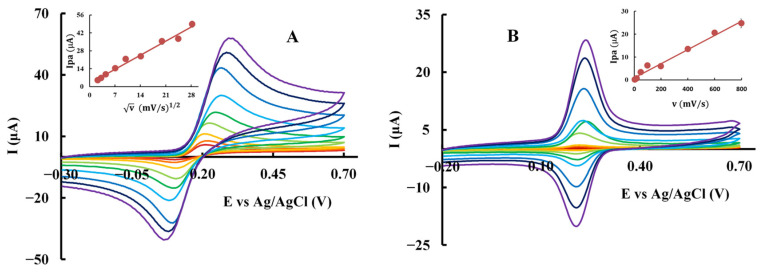
Overlay of cyclic voltammograms at different scan rates: 5, 10, 20, 50, 100, 200, 400, 600, and 800 mV/s obtained in a phosphate buffer (pH 7.4) using a GCE for (**A**) 1 mM of CA; inset: *Ipa* vs. *v*^1/2^. (**B**) 1 mM of CA-P; inset: *Ipa* vs. *v*.

Further studies were conducted to investigate the effect of the scan rate on peak current of phenolic acids and their corresponding esters. An increase in peak current intensity was observed with increasing scan rate, and the data were adjusted to linear regression analysis for both anodic and cathodic processes to elucidate the control mechanism (Figure 3, Table 4).

The Randles–Ševčík equation, which describes the relationship between peak current and scan rate for electrochemically reversible electron transfer processes involving freely diffusing redox species, was applied to evaluate the results [24]. This equation predicts a linear relationship between the peak current (*Ip*) and the square root of the scan rate (*v*^1/2^) for a diffusion-controlled process (Figure 3A). The results confirmed that all phenolic acids followed the expected diffusion-controlled behavior. In contrast, the phytyl esters exhibited a direct correlation between *Ip* and *v* (Figure 3B), indicating that their electrochemical process is predominantly controlled by an adsorption mechanism. The linear regressions obtained demonstrated a strong correlation between *Ip* and either *v^1/2^* or *v*, with a high correlation coefficient (Table 4). A visual analysis of the overlay of cyclic voltammograms at different scan rates also complements the conclusions made (Figure 3). The shape and symmetry of the anodic peak provide additional evidence to distinguish between diffusion and adsorption-controlled processes.

**Figure 4:**
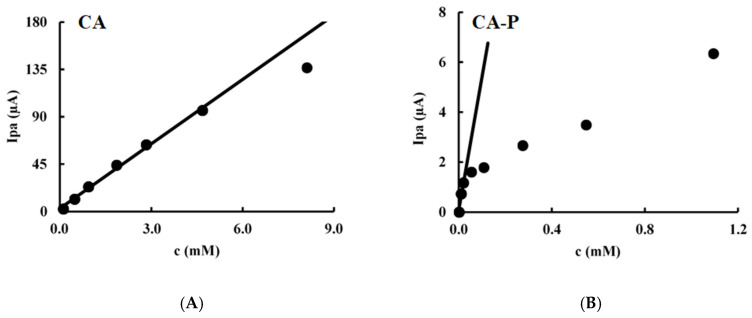
Effect of concentration on Ip at 100 mV/s obtained in a phosphate buffer (pH 7.4) using a GCE for (**A**) CA and (**B**) CA-P.

The effect of concentration variation on *Ipa* and *Epa* was studied over a range of 0.1 mM to 5 mM for CA and 0.01 mM to 1 mM for its ester (CA-P), with the upper limit for the ester being reached by its solubility (Figure 4).

In diffusion-controlled processes, the anodic peak current increases proportionally with concentration as the analyte is freely diffusing in solution. The linearity of the *Ip* vs. concentration plot up to 5 mM is more pronounced for CA than for its ester (Figure 4). This behavior is not observed in the adsorption-controlled process for CA-P, where the response is primarily limited by the available electrode surface area. For CA, a diffusion-controlled compound, a proportional relationship was established between peak current and concentration, as well as between anodic peak potential and the logarithm of the concentration.

In the case of CA-P, which follows an adsorption-controlled mechanism, the proportionality between peak current and concentration was only observed up to 0.055 mM. (Figure 4). Beyond this concentration, oxidation is limited by electrode surface saturation. This behavior arises from the fact that the molecules first adsorb onto the electrode surface before undergoing oxidation. Once the available surface sites are saturated, no further increase in peak current is observed, regardless of higher analyte concentrations in solution. In relation to the variation of potential with concentration, it was observed that the potential required for oxidation remained constant for the phytyl ester (*Epa* = 216 ± 1 mV). Since the molecules are pre-adsorbed onto the electrode surface, the oxidation potential remains largely unaffected by increasing the analyte concentration.

The electrochemical characterization of molecules provides valuable insights for optimizing their application across different fields. Oxidation mechanisms could significantly influence the antioxidant efficiency depending on the system in which they are applied. Therefore, compounds that undergo diffusion-controlled oxidation could be suitable for use in homogeneous liquid systems, whereas those that easily adsorb can be particularly effective in lipid-based systems where surface interactions are crucial for protection against oxidative stress.

### 2.3. Interaction of Compounds with Liposomal Membranes

Esterifying phenolic acids with phytol enhances their liposolubility, as indicated by miLog *p* values, bringing them closer to that of α-Toc (Table 2). This increase in liposolubility suggests an improved ability for these phenolipids to interact with biomembranes. To investigate how these phenolipids interact with lipid bilayers, two simplified biomembrane models were employed. Liposomes made of 1,2-dimyristoyl-sn-glycero-3-phosphocholine (DMPC) were prepared and incorporated with three different fluorescent membrane probes—2-hydroxyacid-(9-anthroyloxy)stearic acid (2-AS), 1,6-diphenyl-1,3,5-hexatriene-4′-trimethylammonium tosylate (TMA-DPH), and 1,6-diphenyl-1,3,5-hexatriene (DPH)—which localize at various depths from the bilayer center (16, 10.9, and 7.8 Å, respectively) [25].

**Figure 5:**
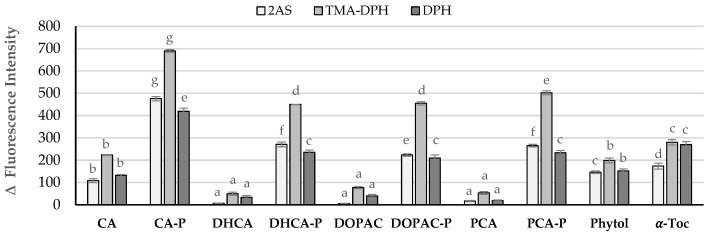
Quenching effect of AOs on 2-AS, TMA-DPH, and DPH probe emission intensity using DMPC liposomes. Error bars represent standard deviation. Different letters for each probe represent statistically different means, *p* < 0.05.

The quenching of the fluorescence emission of probes when the AOs were present (Figure 5) revealed that the parental phenolic acids have a low effect on the three fluorescence probes, indicating a more external localization in the membrane. The exception was caffeic acid (CA), which showed a similar ability to phytol to penetrate the membrane, likely due to its higher lipophilicity. Although the miLog *p* values—representing the estimated partition between water and n-octanol—are quite similar for the various parental phenolic acids, the actual partition coefficient between water and vegetable oils differs significantly in the case of caffeic acid [26]. In fact, CA exhibits much lower solubility in water compared to the other phenolic acids, with oil–water partition coefficients around 0.5, in contrast to the values in the range of −0.1 to −0.2 typically observed for the other parental acids. This suggests that the enhanced membrane penetration of CA may be better explained by its oil–water partitioning behavior than by its predicted octanol–water Log *p* value. Although Log *p* is widely used in medicinal chemistry as a standard indicator of molecular lipophilicity, particularly in drug design, it may not always accurately reflect membrane interactions in biological or lipid-rich environments. Additionally, the presence of the trans double bond in the side chain of caffeic acid may also contribute to its behavior, as it confers greater linearity and rigidity to the molecule. This structural feature could facilitate a more favorable alignment within the lipid bilayer, thereby enhancing membrane interaction.

**Figure 6:**
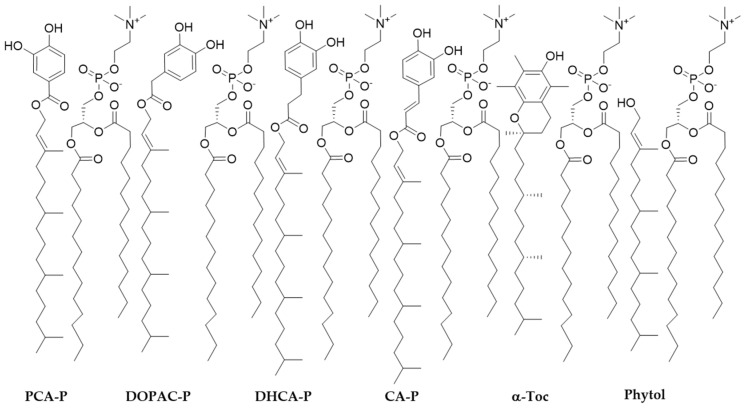
Representative arrangement of phytyl esters embedded in the phosphatidylcholine membrane monolayer.

In contrast, the fluorescence intensity of the three probes decreased sharply within a few seconds after the addition of phytyl esters or α-tocopherol into the DMPC liposome suspension, showing interactions of the compounds along the membrane monolayer (Figure 5). These interactions indicate that these molecules are found in a parallel position to the phospholipid molecules (Figure 6).

However, the interaction of compounds with the probes did not display the same intensity, with CA-P showing the highest interaction. The quenching observed in the TMA-DPH fluorescence intensity is higher for the phytyl esters than with α-tocopherol, showing a strong interaction of the compounds with the area of the bilayer below the phospholipids polar head. However, in the case of the DPH probe, only CA-P showed a higher quenching capacity when compared to α-Toc and to the other esters, which may indicate, for this ester, a deeper location in the monolayer.

**Figure 7:**
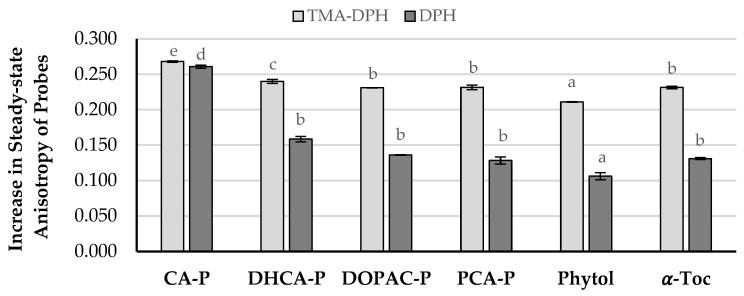
Increase in the steady-state anisotropy of probes TMA-DPH and DPH in DMPC liposomes. Error bars represent standard deviation. Different letters for each probe represent statistically different means, *p* < 0.05.

The influence of phytyl compounds in the DMPC LUVs model bilayer fluidity was also evaluated at 37 ± 0.1 °C and compared with that of α-Toc. Figure 7 shows changes in the steady-state fluorescence anisotropy of the DPH and TMA-DPH probes in the presence of compounds. The results show that, at the concentration tested, phytyl phenolipids increase the anisotropy values of both probes, either located in the intermediate and in the bottom regions of the monolayer, showing that all compounds decreased the fluidity of the membrane in both regions. Besides the higher quenching capacity observed in the TMA-DPH fluorescence intensity for the phytyl esters, the decrease in the anisotropy values of both probes was like that obtained for α-Toc except for CA-P, indicating a low probability of these compounds to present toxicity caused by interference in membrane fluidity. In contrast, CA-P, when compared to α-tocopherol, was able to significantly change the steady-state anisotropy parameter for the DPH probe, resulting in a lower fluidity of the membrane in this region [16] and confirming its deeper location in the monolayer.

### 2.4. Antioxidant Capacity of Phytyl Phenolipids in Liposomal Systems

**Figure 8:**
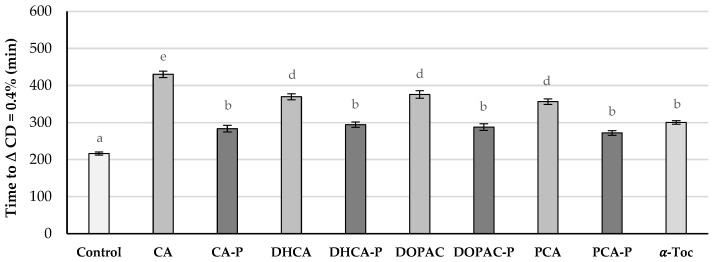
Time in minutes required for liposomes incubated at 37 °C without (control) and with AOs at 0.75 μM in the presence of AAPH to reach a conjugated diene (CD) content of 0.4%. Error bars represent standard deviation. Different letters represent statistically different means, *p* < 0.05.

In this study, AAPH, a water-soluble free radical initiator widely used in biological studies was used. This azo compound reacts with oxygen, producing peroxyl radicals at a constant rate, and it is intend to mimic the attack of free radicals such as hydroperoxyl and peroxyl radicals to biomembranes [25]. In the presence of AOs, the oxidative stability of liposomal suspensions of lecithin from soybean (PC soy) containing AAPH increased when compared with the control (Figure 7) for all parental compounds and phytyl esters. Nevertheless, the antioxidant efficiency of parental phenolic acids proved to be better than that of phytyl derivatives or α-Toc. The antioxidant efficiency order was CA > DHCA ~ DOPAC ~ PCA > α-Toc ~ CA-P ~ DHCA-P ~ DOPAC-P ~ PCA-P > Control (Figure 8).

Despite the lower anodic potential and the higher interaction with the bilayer found for these compounds, a better antioxidant capacity was not observed for the phytyl esters when compared to the parental compounds. Still, phytyl esters showed similar antioxidant protection to that of α-Toc. Similar results were already obtained for cholesteryl phenolipids [13] and other more lipophilic phenolipids [16]. Due to their high liposolubility (Table 1), these compounds, when in contact with PBS, may form aggregates and hinder their penetration into the membrane. Therefore, the stochiometric concentration can be much higher than the actual concentration of esters in the bilayer, leading to a relatively low antioxidant capacity. Another reason can be the fact that AAPH-derived radicals are formed in the aqueous media, and these radicals may be more efficiently scavenged by hydrophilic compounds before the attack to the membrane.

**Figure 9:**
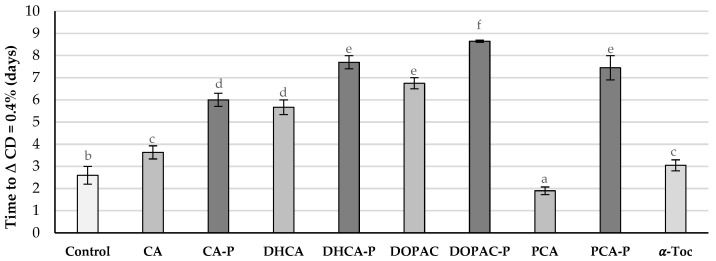
Time in days required for liposomes incubated at 37 °C without (control) and with AOs at 0.75 μM to reach a conjugated diene (CD) content of 0.4%. Error bars represent standard deviation. Different letters represent statistically different means, *p* < 0.05.

A further oxidative antioxidant assay was executed, this time without the use of a radical initiator (Figure 9). Liposomes were incubated in the presence of compounds and stored in the dark at 37 °C, and the contents in conjugated dienes were followed by UV spectrophotometry.

With this procedure, free radicals are formed inside the membrane and will migrate to the polar head zone of the membrane [26]. In the presence of AOs, the oxidative stability against autoxidation increased for most suspensions (Figure 8) except for the ones containing PCA, being now the order of antioxidant activity different to that obtained in the previous stability study: DOPAC-P > DHCA-P > PCA-P ~ DOPAC > CA-P ~ DHCA > CA ~ α-Toc > Control > PCA. Under the conditions of this study, the liposomal suspensions containing phytyl esters showed more than twice the stability of the liposomes containing α-Toc. These results show that the lipophilization of phenolic acids with the phytyl moiety increased their antioxidant efficiency. In the case of PCA, this increase was very significant as the parental compound showed pro-oxidant effects. This pro-oxidant activity is common in lipidic systems for the more hydrophilic antioxidants and is related to the interactions of these reducing molecules with metal traces present in the system [27]. The increase in the efficiency of this compound may also be related to the increase in its radical scavenging capacity and the lowering of its anodic potential. Differences in the antioxidant efficiency among the phytyl esters are mostly explained by the higher or lower availability of the aromatic hydroxy groups to act as radical scavengers near the polar phospholipid heads. CA-P showed the worst antioxidant capacity among the phytyl esters probably because it may be located slightly deeper in the membrane, confirmed by the higher interference of this compound in the membrane fluidity near the DPH probe when compared to the other phytyl esters. Relatively small differences in the structure of antioxidants may interfere with their capacity in protecting membranes because of their different locations. This can be observed for tocopherols and tocotrienols. In fact, these compounds show similar radical scavenging capacity against several free radicals when in solution, but their capacities in protecting biomembranes are quite different, being usually α-tocopherol, as a result of its better location/interaction within the membrane, the one with the highest efficiency [28].

## 3. Materials and Methods

### 3.1. Materials

Commercially available phytol (97%) containing a mixture of E and Z diastereoisomers in the proportion of 3:1, respectively (checked by NMR, and in accordance with the NMR spectra given by the supplier), was obtained from Sigma-Aldrich and was used without any previous purification. CA, 3,4-dihydroxybenzaldeyde, β-Alanine (99%), DPPH^•^ (95%), and DPH were also acquired from Sigma-Aldrich. α-Toc (95%), PCA (97%), and thionyl chloride (99.7%) were purchased from Thermo Fisher Scientific. Meldrum’s acid (>98%) and DOPAC (>98%) were sourced from TCI, while pyridine (99.5%) was acquired from Merck. PC Soy (90%) and anhydrous sodium sulfate (99.9%) came from Panreac AppliChem. Hydrochloric acid (37%) and TMA-DPH were purchased from Fluka. DHCA (>98%), 2-AS, and DMPC were supplied by Alfa Aesar, Molecular Probes, and Avanti Polar Lipids, respectively. Enzyme 435 was obtained from Novozymes. All organic solvents used were of analytical or HPLC grade and were purchased from Fisher Scientific.

### 3.2. Synthesis of Phytyl Esters of Polyphenolic Acids

All syntheses and purifications were monitored using Thin-Layer Chromatography (TLC) on silica gel plates (FluoroChem, Hadfield, UK) 60 F254 on aluminum support and petroleum ether/ethyl acetate (3:1, *v*/*v*) as eluent, with development in an iodine chamber.

Syntheses of phytyl esters were further followed by High-Performance Liquid Chromatography (HPLC) using a Vanquish equipment from Thermoscientific (Lisboa, Portugal), with a Diode-array detector and Chromeleon 7 software. It was used a LiChrospher^®^ 100 RP-18 (5 μm) LiChroCART^®^ 250-4 reverse-phase column and a LiChrospher^®^ 100 RP-18 (5 μm) LiChroCART^®^ 4-4 precolumn, both from Merck. The sample compartment and column temperatures were set to 30 °C. Then, 10 μL of each sample was analyzed for 30 min using ACN/MeOH (1:1, *v*/*v*) as an eluent at a flow rate of 1.0 mL/min with UV detection at 280 nm. Before analyzing, samples were filtered using a 0.22 μm polyvinylidene fluoride (PVDF) syringe filters. The retention time (RT) and the maximum absorption wavelength (λ_max_) of each compound were determined using HPLC. A mixture of diastereoisomers was obtained in all syntheses in the same proportion found in the commercially available phytol (3E:1Z).

The synthetized compounds were identified and characterized by Proton and Carbon Nuclear Magnetic Resonance (^1^H-NMR and ^13^C-NMR, respectively), using deuterated chloroform (VWR) as solvent. NMR spectra were acquired on a Bruker Advance III NMR spectrometer at the Centre for Materials of the University of Porto (CEMUP) and analyzed using MestReNova v14.2.3-29241 software. Chemical shifts (δ) and coupling constants (J) are reported in parts per million (ppm) and Hertz (Hz), respectively.

The exact mass of the new phytyl esters was confirmed by High-Resolution Mass Spectroscopy (HRMS) on an Orbitrap Exploris 120 mass spectrometer (Thermo Fischer Scientific, Bremen, Germany) controlled by Orbitrap Exploris Tune Application 2.0.185.35 and Xcalibur 4.4.16.14 software. The resolution of SIM MS scan was 60,000.

#### 3.2.1. Synthesis of CA-P

CA-P was synthesized following a two-step protocol as previously described [13]. In the first step, phytyl monomalonate was synthesized using Meldrum’s acid. The obtained monomalonate underwent a Knoevenagel condensation with 3,4-dihydroxybenzaldehyde to produce phytyl caffeate.

In summary, equimolar amounts (3.4 mmol) of Meldrum’s acid and phytol were refluxed for 4 h in 6 mL of toluene. After evaporation of the solvent, phytyl monomalonate was obtained as a yellow oil (~96%, Rf = 0.68; RT = 6.12 min (Z), 6.42 min (E); UV-Vis (MeOH) λ_max_ 203, 215, 245, 325 nm), which was directly used in the synthesis of the caffeate without further purification. ^1^H-NMR (400 MHz, CDCl_3_): δ 5.34 (m, 1H, H-2); 4.70 (d, *J*_1(E),2_ = 8.0 Hz, 1H, H-1, E); 4.66 (d, *J*_1(Z),2_ = 8.8 Hz, 1H, H-1, Z); 3.44 (s, 2H, H-22, E); 3.43 (s, 2H, H-22, Z); 2.04 (m, 2H, H-5); 1.76 (d, *J*_4(Z),2_ = 1.3 Hz, 3H, H-4, Z); 1.70 (d, *J*_4(E),2_ = 1.3 Hz, 3H, H-4, E); 1.52 (m, 1H, H-18); 1.24 (m, 18H, H-6–H-8, H-10–H-13, H-15–H-17); 0.85 (m, 12H, H-9, H-14, H-19, H-20) ppm. ^13^C-NMR (100 MHz, CDCl_3_): δ 169.90 (C-21); 167.90 (C-23); 144.71 (C-3, Z); 144.41 (C-3, E); 117.89 (C-2, Z); 117.08 (C-2, E); 63.15 (C-1, E); 62.84 (C-1, Z); 40.31 (C-5); 40.00 (C-17); 39.51 (C-22); 37.57 (C-7, E); 37.53 (C-10, E); 37.49 (C-12, E); 37.44 (C-15, E); 37.06 (C-7, Z); 36.96 (C-10, Z); 36.88 (C-12, Z); 36.78 (C-15, Z); 32.93 (C-8, E); 32.80 (C-8, Z); 32.51 (C-13); 28.13 (C-18); 25.75 (C-6, Z); 25.16 (C-11, Z); 24.94 (C-6, E); 24.61 (C-11, E); 23.64 (C-16); 22.86 (C-19); 22.77 (C-20); 19.89 (C-9, E); 19.83 (C-14, E); 19.78 (C-9, Z); 19.73 (C-14, Z); 16.57 (C-4).

CA-P was synthesized by reacting the prepared monomalonate with 3,4-dihydroxybenzaldehyde through a Knoevenagel condensation. Equimolar amounts (4.6 mmol) of monomalonate and 3,4-dihydroxybenzaldehyde were combined in 10.0 mL of pyridine, along with 50 mg of β-alanine, and heated at 50 °C for 5 days. After the reaction, the mixture was rapidly cooled, and 15 mL of concentrated HCl was added. The mixture was then treated with 80 mL of deionized water, and the aqueous phase was extracted three times with 50 mL portions of diethyl ether. The organic phase was dried over anhydrous Na2SO4, and the solvent was subsequently evaporated. The final compound was purified using flash column chromatography over silica gel with petroleum ether/ethyl acetate (3:1, *v*/*v*) as eluent. The CA-P was obtained as a cream-colored solid (~99%; Rf = 0.50; RT = 6.89 min (Z), 7.29 min (E); UV-Vis (MeOH) λ_max_ 205, 215, 245, 300, 325 nm). ^1^H-NMR (400 MHz, CDCl_3_): δ 7.59 (d, *J*_28(E),29_ = 15.9 Hz, 1H, H-28, E); 7.58 (d, *J*_28(Z),29_ = 15.9 Hz, 1H, H-28, Z); 7.09 (d, *J*_22,26_ = 2.1 Hz, 1H, H-22); 7.00 (dd, *J*_26,25_ = 8.2 Hz, *J*_26,22_ = 2.1 Hz, 1H, H-26); 6.87 (d, *J*_25,26_ = 8.2 Hz, 1H, H-25); 6.28 (d, *J*_29(E),28_ = 15.9 Hz, 1H, H-29, E); 6.27 (d, *J*_29(Z),28_ = 15.9 Hz, 1H, H-29, Z); 6.12 (s, 1H, -OH); 5.95 (s, 1H, -OH); 5.40 (tq, *J*_2(E),1_ = 7.1 Hz, *J*_2(E),4_ = 1.4 Hz, 1H, H-2, E); 4.73 (d, *J*_1(E),2_ = 7.1 Hz, 1H, H-1, E); 4.70 (d, *J*_1(Z),2_ = 7.2 Hz, 1H, H-1, Z); 2.11 (t, *J*_5(Z),6_ = 8.2 Hz, 2H, H-5, Z); 2.02 (t, *J*_5(E),6_ = 7.5 Hz, 2H, H-5, E); 1.77 (d, *J*_4(Z),2_ = 1.4 Hz, 3H, H-4, Z); 1.73 (d, *J*_4(E),2_ = 1.4 Hz, 3H, H-4, E); 1.52 (m, 1H, H-18); 1.22 (m, 18H, H-6–H-8, H-10–H-13, H-15–H-17); 0.85 (m, 12H, H-9, H-14, H-19, H-20) ppm. ^13^C-NMR (100 MHz, CDCl_3_): δ 168.08 (C-21, E); 168.02 (C-21, Z); 146.52 (C-23); 145.11 (C-28); 143.98 (C-3, E); 143.70 (C-3, Z); 143.21 (C-24); 127.72 (C-27); 122.54 (C-26); 118.90 (C-2, Z); 118.10 (C-2, E); 115.81 (C-29); 115.64 (C-25); 114.57 (C-22); 61.84 (C-1, E); 61.47 (C-1, Z); 40.06 (C-5); 39.53 (C-17); 37.59 (C-7, E); 37.54 (C-10, E); 37.48 (C-12, E); 37.45 (C-15, E); 37.10 (C-7, Z); 37.01 (C-10, Z); 36.92 (C-12, Z); 36.83 (C-15, Z); 32.93 (C-8, E); 32.88 (C-8, Z); 32.83 (C-13, E); 32.56 (C-13, Z); 28.13 (C-18); 25.80 (C-6, Z); 25.22 (C-11, Z); 24.95 (C-6, E); 24.62 (C-11, E); 23.67 (C-16); 22.87 (C-19); 22.77 (C-20); 19.90 (C-9, E); 19.87 (C-9, Z); 19.81 (C-14, E); 19.75 (C-14, Z); 16.60 (C-4). HRMS (ESI): *m*/*z* calculated for C_29_H_46_O_4_ = 458.3396; found = 458.3299.

#### 3.2.2. Synthesis of DHCA-P and DOPAC-P

DHCA-P and DOPAC-P were synthesized via an esterification reaction using enzymatic catalysis following a previously established procedure. A mixture of 2.8 mmol of DHCA or DOPAC and 8.4 mmol of phytol was dissolved in 30 mL of toluene or dioxane, respectively, along with 1 g of Novozym 435, and the reaction was stirred for 14 days at 55 °C. After the evaporation of the organic solvent, the final phytyl ester was purified using flash column chromatography over silica gel with petroleum ether/ethyl acetate (3:1, *v*/*v*) as eluent.

DHCA-P was obtained as a cream-colored oil (~95%); Rf = 0.50; RT = 6.10 min (Z), 6.38 min (E); UV-Vis (MeOH) λ_max_ 202, 282 nm. ^1^H-NMR (400 MHz, CDCl_3_): δ 6.76 (d, *J*_25,26_ = 8.1 Hz, 1H, H-25); 6.71 (d, *J*_22,26_ = 2.0 Hz, 1H, H-22); 6.62 (dd, *J*_26,25_ = 8.1 Hz, *J*_26,22_ = 2.0 Hz, 1H, H-26); 5.49 (s, 1H, -OH); 5.34 (s, 1H, -OH); 5.31 (tq, *J*_2(E),1_ = 7.1 Hz, *J*_2(E),4_ = 1.4 Hz, 1H, H-2, E); 4.59 (d, *J*_1(E),2_ = 7.1 Hz, 1H, H-1, E); 4.57 (d, *J*_1(Z),2_ = 7.0 Hz, 1H, H-1, Z); 2.83 (t, *J*_28,29_ = 7.7 Hz, 2H, H-28); 2.58 (t, *J*_29(E),28_ = 7.7 Hz, 2H, H-29, E); 2.57 (t, *J*_29(Z),28_ = 7.7 Hz, 2H, H-29, Z); 2.05 (t, *J*_5(Z),6_ = 7.9 Hz, 2H, H-5, Z); 2.00 (t, *J*_5(E),6_ = 8.0 Hz, 2H, H-5, E); 1.75 (d, *J*_4(Z),2_ = 1.3 Hz, 3H, H-4, Z); 1.68 (d, *J*_4(E),2_ = 1.4 Hz, 3H, H-4, E); 1.53 (m, 1H, H-18); 1.22 (m, 18H, H-6–H-8, H-10–H-13, H-15–H-17); 0.85 (m, 12H, H-9, H-14, H-19, H-20) ppm. ^13^C-NMR (100 MHz, CDCl_3_): δ 173.44 (C-21, E); 173.44 (C-21, Z); 143.71 (C-23); 143.50 (C-3, Z); 143.09 (C-3, E); 142.12 (C-24); 133.70 (C-27); 120.78 (C-26); 118.86 (C-2, Z); 118.04 (C-2, E); 115.57 (C-25); 115.49 (C-22); 61.70 (C-1, E); 61.37 (C-1, Z); 40.02 (C-5); 39.52 (C-17); 37.59 (C-7, E); 37.54 (C-10, E); 37.48 (C-12, E); 37.45 (C-15, E); 37.08 (C-7, Z); 36.99 (C-10, Z); 36.92 (C-12, Z); 36.82 (C-15, Z); 36.33 (C-28); 32.94 (C-8, E); 32.87 (C-8, Z); 32.82 (C-13, E); 32.49 (C-13, Z); 31.08 (C-29, Z); 30.49 (C-29, E); 28.13 (C-18); 25.77 (C-6, Z); 25.21 (C-11, Z); 24.95 (C-6, E); 24.62 (C-11, E); 23.62 (C-16); 22.86 (C-19); 22.77 (C-20); 19.90 (C-9, E); 19.86 (C-9, Z); 19.83 (C-14, E); 19.80 (C-14, Z); 16.52 (C-4). HRMS (ESI): *m*/z calculated for C_29_H_48_O_4_ = 460.3553; found = 460.3458.

DOPAC-P was obtained as a cream-colored oil (~83%); Rf = 0.53; RT = 6.15 min (Z), 6.42 min (E); UV-Vis (MeOH) λ_max_ 203, 283 nm. ^1^H-NMR (400 MHz, CDCl_3_): δ 6.78 (d, *J*_22,26_ = 2.0 Hz, 1H, H-22); 6.76 (d, *J*_25,26_ = 8.1 Hz, 1H, H-25); 6.67 (dd, *J*_26,25_ = 8.1 Hz, *J*_26,22_ = 2.0 Hz, 1H, H-26); 5.73 (s, 2H, -OH); 5.33 (tq, *J*_2(E),1_ = 7.2 Hz, *J*_2(E),4_ = 1.4 Hz, 1H, H-2, E); 4.62 (d, *J*_1(E),2_ = 7.2 Hz, 1H, H-1, E); 4.60 (d, *J*_1(Z),2_ = 8.0 Hz, 1H, H-1, Z); 3.50 (s, 2H, H-28); 2.06 (t, *J*_5(Z),6_ = 6.9 Hz, 2H, H-5, Z); 2.00 (t, *J*_5(E),6_ = 7.5 Hz, 2H, H-5, E); 1.75 (d, *J*_4(Z),2_ = 1.4 Hz, 3H, H-4, Z); 1.68 (d, *J*_4(E),2_ = 1.4 Hz, 3H, H-4, E); 1.52 (m, 1H, H-18); 1.22 (m, 18H, H-6–H-8, H-10–H-13, H-15–H-17); 0.85 (m, 12H, H-9, H-14, H-19, H-20) ppm. ^13^C-NMR (100 MHz, CDCl_3_): δ 172.65 (C-21, E); 172.61 (C-21, Z); 143.72 (C-23); 143.57 (C-3, Z); 143.20 (C-3, E); 142.99 (C-24); 126.53 (C-27); 121.78 (C-26); 118.56 (C-2, Z); 117.75 (C-2, E); 116.34 (C-25); 115.37 (C-22); 62.09 (C-1, E); 61.78 (C-1, Z); 40.67 (C-28); 39.89 (C-5); 39.38 (C-17); 37.45 (C-7, E); 37.41 (C-10, E); 37.34 (C-12, E); 37.30 (C-15, E); 36.94 (C-7, Z); 36.85 (C-10, Z); 36.77 (C-12, Z); 36.67 (C-15, Z); 32.83 (C-8, E); 32.78 (C-8, Z); 32.68 (C-13, E); 32.38 (C-13, Z); 27.99 (C-18); 25.62 (C-6, Z); 25.07 (C-11, Z); 24.81 (C-6, E); 24.49 (C-11, E); 22.72 (C-16); 22.63 (C-19); 22.77 (C-20); 19.75 (C-9, E); 19.71 (C-9, Z); 19.70 (C-14, E); 19.65 (C-14, Z); 16.41 (C-4). HRMS (ESI): *m*/*z* calculated for C_28_H_46_O_4_ = 446.3396; found = 446.3309.

#### 3.2.3. Synthesis of PCA-P

PCA-P was synthesized using a one-pot method, as previously outlined [12]. In this process, PCA was first converted to its corresponding acid chloride by reacting with thionyl chloride. The resulting protocatechuate acid chloride was then esterified with phytol to yield the final product. In brief, PCA (6.5 mmol) was dissolved in 30 mL of dichloromethane containing 13 mmol of pyridine, and the solution was stirred while being cooled in an ice bath. Thionyl chloride (13 mmol) was added dropwise to the mixture, and the reaction was allowed to proceed for 24 h. Then, 13 mmol of phytol was added, and the reaction was maintained for 5 days at 4 °C. Finally, after the solvent evaporation, the final compound was purified by flash column chromatography on silica gel using petroleum ether/ethyl acetate (3:1, *v*/*v*) as eluent.

The product was obtained as a cream-colored oil (~70%); Rf = 0.59; RT = 6.53 min (Z), 6.98 min (E); UV-Vis (MeOH) λ_max_ 204, 260, 295 nm; ^1^H-NMR (400 MHz, CDCl_3_): δ 7.65 (d, *J*_22(E),26_ = 2.0 Hz, 1H, H-22, E); 7.65 (d, *J*_22(Z),26_ = 2.0 Hz, 1H, H-22, Z); 7.60 (dd, *J*_26(E),25_ = 8.3 Hz, *J*_26(E),22_ = 2.0 Hz, 1H, H-26, E); 7.60 (dd, *J*_26(Z),25_ = 8.3 Hz, *J*_26(Z),22_ = 2.0 Hz, 1H, H-26, Z); 6.92 (d, *J*_25,26_ = 8.3 Hz, 1H, H-25, E); 6.92 (d, *J*_25(Z),26_ = 8.3 Hz, 1H, H-25, Z); 5.98 (s, 1H, -OH); 5.95 (s, 1H, -OH); 5.46 (tq, *J*_2(E),1_ = 7.0 Hz, *J*_2(E),4_ = 1.3 Hz, 1H, H-2, E); 4.83 (d, *J*_1(E),2_ = 7.0 Hz, 1H, H-1, E); 4.80 (d, *J*_1(Z),2_ = 7.2 Hz, 1H, H-1, Z); 2.15 (t, *J*_5(Z),6_ = 7.6 Hz, 2H, H-5, Z); 2.05 (t, *J*_5(E),6_ = 7.5 Hz, 2H, H-5, E); 1.80 (d, *J*_4(Z),2_ = 1.2 Hz, 3H, H-4, Z); 1.77 (d, *J*_4(E),2_ = 1.3 Hz, 3H, H-4, E); 1.54 (m, 1H, H-18); 1.25 (m, 18H, H-6–H-8, H-10–H-13, H-15–H-17); 0.88 (m, 12H, H-9, H-14, H-19, H-20) ppm. ^13^C-NMR (100 MHz, CDCl_3_): δ 166.80 (C-21); 148.67 (C-23); 143.57 (C-24); 143.04 (C-3); 124.04 (C-26); 123.23 (C-27); 119.03 (C-2, Z); 118.25 (C-2, E); 116.81 (C-25); 114.97 (C-22); 62.08 (C-1, E); 61.71 (C-1, Z); 40.05 (C-5); 39.53 (C-17); 37.59 (C-7, E); 37.54 (C-10, E); 37.48 (C-12, E); 37.45 (C-15, E); 37.10 (C-7, Z); 37.03 (C-10, Z); 36.92 (C-12, Z); 36.82 (C-15, Z); 32.94 (C-8, E); 32.90 (C-8, Z); 32.82 (C-13, E); 32.59 (C-13, Z); 28.13 (C-18); 25.82 (C-6, Z); 25.23 (C-11, Z); 24.95 (C-6, E); 24.62 (C-11, E); 23.65 (C-16); 22.87 (C-19); 22.77 (C-20); 19.89 (C-9, E); 19.85 (C-9, Z); 19.81 (C-14, E); 19.75 (C-14, Z); 16.63 (C-4). HRMS (ESI): *m*/*z* calculated for C_27_H_44_O_4_ = 432.3240; found = 432.2504.

### 3.3. Determination of miLog p Values

The Molinspiration property engine v2022.08 (Molinspiration Cheminformatics, accessed on 4 January 2025) was used to estimate the lipophilicity of the phytyl compounds by calculating the miLog *p* values.

### 3.4. DPPH Radical Scavenging Capacity

The radical scavenging activity of the compounds was assessed using the DPPH• scavenging capacity assay following methods previously outlined [13]. In short, 50 μL of the compound stock solutions in ethanol, at varying final concentrations (3–25 μM), were added to each well of a 96-well microplate. Then, 250 μL of a DPPH• solution (final concentration 112 μM) was added to each well to start the reaction, and absorbance was measured at 5-min intervals over a 30 min period at 25 °C. The free radical scavenging activity was quantified by determining the EC_50_ value, which represents the concentration of the antioxidant required to reduce the initial DPPH• concentration by 50%. Absorbance readings taken at 5 and 30 min were used to calculate the EC_50_ values. Each compound was tested in duplicate across five independent experiments.

### 3.5. Cyclic Voltammetry

Voltammetric measurements were carried out using an Autolab PGSTAT 30 potentiostat (Eco-Chemie) (Utrecht, The Netherlands) controlled by the GPES 4.9 software package (General Purpose Electrochemical Experiments). The experiments were performed in a three-electrode configuration, where a glassy carbon electrode (GCE) served as the working electrode, a platinum wire was used as the counter electrode, and an Ag/AgCl (1 M KCl) electrode acted as the reference. Prior to each measurement, the working electrode was polished with 0.05 µm alumina powder on a polishing cloth and rinsed with water. The scan rates used were 5, 10, 20, 50, 100, 200, 400, 600, and 800 mV/s.

To prepare solutions for the evaluation of peak potential, 100 µL of 0.10 M antioxidant solution prepared in ethanol was transferred to a 10 mL volumetric flask, and the volume was made up with phosphate buffer (pH 7.4). The solutions prepared for the voltammetric characterization were all nominally 1 mM. However, their actual concentrations showed slight variations: 0.94 mM for CA, 1.00 mM for DHCA, 1.04 mM for DOPAC, 1.04 mM for PCA, 1.10 mM for CA-P, 0.86 mM for DHCA-P, 0.95 mM for DOPAC-P, 0.60 mM for PCA-P, and 1.34 mM for α-Toc. The effect of the concentration was studied for CA at 0.1, 0.5, 1, 2, 4, and 5 mM, and for its phytyl ester CA-P at 0.01, 0.02, 0.05, 0.1, 0.3, 0.5, and 1 mM.

All mean values and statistical parameters of the regression line are reported with their standard deviations calculated from a set of three consistent replicates.

### 3.6. Preparation of Large Unilamellar Vesicles

Liposomes were utilized as membrane-mimicking systems to evaluate the antioxidant activity of the compounds and their integration into the membrane. The compounds were either incubated with pre-formed liposomes or added during the liposome formation process, also referred to as phytosomes.

The preparation of liposome suspensions followed the hydration-extrusion method described previously [15]. In brief, PC soy or DMPC lipids were dissolved in chloroform in a round-bottom flask, and the solvent was removed by evaporation, leaving behind a lipid film. The film was then subjected to a vacuum for 3 h. To hydrate the lipid film, PBS (0.01 M, pH 7.4) was added, resulting in the formation of large multilamellar vesicles (MLVs). These MLVs were extruded through a 100 nm polycarbonate filter (Whatman, Merck, Lisbon, Portugal) using a LIPEX Biomembrane extruder at 37 °C for 10 cycles to produce large unilamellar vesicles (LUVs) with a diameter of 100 nm. The prepared LUV suspension was stored at 4 °C until further use.

For quenching assays, fluorescence probes were incorporated into the liposomes by adding each probe to the initial DMPC chloroform solution, maintaining a probe–lipid ratio of 1:300 for DPH or TMA-DPH and 1:100 for 2-AS.

For anisotropy assays, antioxidants (AOs) and fluorescence probes (DPH or TMA-DPH) were incorporated into the liposomes by adding each probe at a ratio of 1:300, along with each AO in an ethanolic solution (final concentration, 200 µM), to the initial DMPC chloroform solution.

### 3.7. Dynamic Light Scattering Measurements

To determine the average size and polydispersity index (P.I.) of liposome suspensions, dynamic light scattering (DLS) was employed [12] using a Zetasizer Nano-ZS instrument (Malvern Instruments, version 7.12, Malvern, UK). The samples were analyzed by backscattering with a helium–neon laser (633 nm) at a 173° angle using refractive indices of 1.330 for PBS (water) and 1.400 for lipids, and a dispersant viscosity of 0.6913 cP (water), at 37 °C. LUV preparations with an average diameter of 112 ± 2 nm were obtained, exhibiting inter-day variability below 5% and a polydispersity index (P.I.) of less than 0.1.

### 3.8. Fluorescence Quenching Measurements

The fluorescence quenching assay was employed to assess the incorporation of both the parent and synthesized compounds into the LUV membrane using three fluorescence probes (DPH, TMA-DPH, and 2-AS) [15,29,30]. α-Tocopherol served as the reference compound. The assays were performed on a Varian Cary Eclipse spectrofluorometer with a 1 cm quartz cuvette. The excitation and emission wavelengths for DPH, TMA-DPH, and 2-AS were 360/427 nm, 365/426 nm, and 363/458 nm, respectively, with both excitation and emission slit widths set to 5 nm and a scan speed of 120 nm/min with 1 nm data intervals.

The DMPC LUV suspension at 1 mM, containing the respective probe, was first incubated at 37 °C to achieve thermal equilibrium. Fluorescence quenching measurements began at time 0, and after 4 min, 20 µL of AO solution (final concentration of 200 µM) was added. Fluorescence intensity was recorded over the subsequent 26 min. Each compound and probe combination were measured independently three times.

### 3.9. Effect of Compounds on the Fluorescence Polarization of Probes

Fluorescence anisotropy was measured using two fluorescence probes (DPH and TMA-DPH) and antioxidants (AOs) incorporated into the LUVs at a concentration of 200 µM, following the method described previously [12]. The measurements were carried out on a Varian Cary Eclipse spectrofluorometer with a 1 cm quartz cuvette. For DPH and TMA-DPH, excitation and emission wavelengths of 360/427 nm and 365/426 nm, respectively, were used, with slit widths of 5 nm for both excitation and emission. The steady-state anisotropy <r> values were calculated based on the formula outlined in [31]:

$$<r>=\frac{I_{VV}-I_{VH}G}{I_{VV}+2\;I_{VH}G}$$

Here, *I_VV_* and *I_VH_* refer to the emission intensities when the emission polarizer is oriented vertically (parallel) and horizontally (perpendicular) to the polarization of the excitation light, respectively. G is a correction factor, calculated as the ratio of the vertical to horizontal components when the excitation light is polarized in the horizontal direction, $G=I_{HV}/I_{HH}$. The anisotropy values are then expressed as the mean of the values of two independent assays.

### 3.10. Evaluation of the Antioxidant Activity of Compounds in PC Liposomes

The antioxidant efficiency of phytyl esters and their parent phenolic acids was assessed in PC soy LUVs [12], both with and without the addition of the free radical initiator AAPH in PBS (final concentration of 0.40 µM). To begin, 250 µL of PC soy LUVs (final concentration of 175 µM) was added to a UV-Star™ 96-well microplate (Greiner Bio-one Merck, Lisbon, Portugal). Next, 20 µL of the DMSO solution of each phenol (final concentration 0.75 µM) was added to the LUVs, and the mixture was incubated at 37 °C. The increase in conjugated diene concentration was monitored by measuring the absorbance of each sample at [12] 4 nm at 5 min intervals over a 10 h period when the radical initiator was added, and daily over a 10-day period when the initiator was not included, using a Powerwave XS Microplate Reader (Bio-Tek Instruments, Soquimica, Lisbon, Portugal) [16,32]. Microplates were sealed during storage to prevent water evaporation.

α-Tocopherol, a known natural biomembrane AO, was tested as a reference. The results are presented as the time (in minutes or hours) required for a 0.4% increase in the conjugated diene concentration for each sample. Each compound was tested in triplicate across four independent experiments with the presence of AAPH and in triplicate in two independent experiments without the presence of AAPH.

### 3.11. Statistical Analysis

Statistical analysis was performed using SPSS 29.0 software. One-way analysis of variance (ANOVA) with Duncan’s test was used, with the level of significance was set at *p* < 0.05.

## 4. Conclusions

In this study, methods were developed for the straightforward synthesis of a novel series of phenolipids, resulting in high yields (70–95%) of phytyl esters of polyphenolic acids. Preliminary characterization of the new compounds revealed that they exhibit similar liposolubility and comparable radical scavenging activity to the most important natural membrane antioxidant α-tocopherol. Cyclic voltammetry demonstrated lower anodic peak potentials for the phytyl esters compared to their parent phenolic acids. The results indicate that all phenolic acids exert their activity via an electron transfer mechanism controlled by diffusion. In contrast, the phytyl esters follow an adsorption-controlled mechanism, which is particularly relevant in lipid-based systems where surface interactions are key for protection against oxidative stress. Despite their strong anti-radical capacity and ability to penetrate membranes, the antioxidant effectiveness of the phytyl esters in LUVs was also dependent on the structure of the polyphenolic moiety of the phenolipids. In the PC soy LUVs system tested, DOPAC-P was the most effective in preventing liposome autoxidation. As all the phytyl esters showed better efficiency when compared to α-tocopherol, all these molecules can be considered promising candidates for future interventions against in vivo oxidative stress, deserving a proper evaluation of their antioxidant and anti-inflammatory capacity in biological models. Furthermore, phytol has itself demonstrated a broad spectrum of bioactivities, including antimicrobial activity, antianxiety, immune-modulating effects, and reduction of oxidative stress in cell models [19,29,33,34], and, therefore, its derivatives are of particular interest in developing treatments for chronic diseases [30,31,35] and antiaging formulations [36]. In conclusion, phytyl phenolipids are promising candidates for antioxidant protection of biomembranes, conferring higher efficiency than the natural antioxidant α-tocopherol.

## Figures and Tables

**Table 1:** Yields obtained for the synthesis of phytol esters through acid and enzymatic catalysis using different organic solvents. The values were obtained through HPLC analysis (NR—no reaction).

Compound	Acid Catalysis		Enzymatic Catalysis			
	Toluene	THF	Toluene	Dioxane	DCM	THF
DCA-P	NR	17%	95%	50%	NR	NR
DOPAC-P	NR	56%	46%	83%	NR	NR
PCA-P	NR	NR	18%	0%	NR	NR

**Table 2:** EC_50_ (mol compound/mol DPPH^●^) and miLog *p* values *.

Compound	miLog *p*	EC_50_ **		Compound	miLog *p*	EC_50_ **	
		5 min	30 min			5 min	30 min
Phytol	6.76	-	-	α-Toc	9.04	0.33	0.29
PCA	0.86	0.22	0.19	PCA-P	8.20	0.19	0.14
DOPAC	0.39	0.13	0.13	DOPAC-P	8.09	0.21	0.18
DHCA	0.91	0.19	0.13	DHCA-P	8.26	0.29	0.29
CA	0.94	0.23	0.21	CA-P	8.49	0.23	0.22

* Data Log *p* obtained from Molinspiration Cheminformatics. ** Standard deviations were lower than 5%.

**Table 3:** Anodic peak potential (*Epa*), cathodic peak potential (*Epc*), anodic peak current (*Ipa*), and cathodic peak current (*Ipc*) of phenolic acids and their corresponding phytyl esters.

Compound	*Epa* (V) *	*Epc* (V) *	*Ipa* (µA) *	*−Ipc* (µA) *	Compound	*Epa* (V) *	*Epc* (V) *	*Ipa* (µA) *	*−Ipc* (µA) *
CA	0.223	0.091	21.67	17.79	CA-P	0.235	0.198	6.71	2.25
DHCA	0.297	−0.026	17.31	14.63	DHCA-P	0.250	0.171	4.49	4.22
DOPAC	0.403	0.053	14.67	12.72	DOPAC-P	0.219	-	4.56	-
PCA	0.387	0.082	15.18	10.35	PCA-P	0.327	0.303	4.55	0.35
					α-Toc	0.219	-	4.10	-

* Standard deviations were lower than 5%.

**Table 4:** Linear regression equation and correlation coefficient, r, used to analyze the process controlling the electrochemical reaction, where *Ip* is in µA and *v* in mV/s.

Compound	Anodic Process		Cathodic Process		Controlled by
	Linear Regression Equation	*r*	Linear Regression Equation	*r*	
CA	*Ip* = (1.57 ± 0.08) *v*^1/2^ + (2.6 ± 1.2)	0.991	*−Ip* = (1.63 ± 0.07) *v*^1/2^ + (−0.6 ± 1.2)	0.993	Diffusion
DHCA	*Ip* = (1.54 ± 0.04) *v*^1/2^ + (2.0 ± 0.6)	0.998	*−Ip* = (1.23 ± 0.09) *v*^1/2^ + (1.0 ± 1.4)	0.980	Diffusion
PCA	*Ip* = (1.43 ± 0.01) *v*^1/2^ + (1.0 ± 0.2)	0.999	*−Ip* = (1.25 ± 0.03) *v*^1/2^ + (−0.9 ± 0.5)	0.998	Diffusion
DOPAC	*Ip* = (1.30 ± 0.02) *v*^1/2^ + (2.1 ± 0.3)	0.999	*−Ip* = (1.20 ± 0.04) *v*^1/2^ + (−1.5 ± 0.7)	0.995	Diffusion
CA-P	*Ip* = (0.031 ± 0.002) *v* + (0.9 ± 0.6)	0.991	*−Ip* = (0.0231 ± 0.0004) *v* + (−0.2 ± 0.1)	0.999	Adsorption
DHCA-P	*Ip* = (0.0388 ± 0.0006) *v* + (0.8 ± 0.1)	0.999	*−Ip* = (0.054 ± 0.001) *v* + (−0.8 ± 0.4)	0.997	Adsorption
PCA-P	*Ip* = (0.030 ± 0.001) *v* + (0.7 ± 0.4)	0.994	*−Ip* = (0.0125 ± 0.0006) *v* + (−0.5 ± 0.2)	0.993	Adsorption
DOPAC-P	*Ip* = (0.0251 ± 0.0009) *v* + (1.0 ± 0.3)	0.996	-	-	Adsorption

## Data Availability

Data are contained within the article.
